# The Heart Renaissance

**DOI:** 10.31083/j.rcm2503091

**Published:** 2024-03-06

**Authors:** Vincent Michael Figueredo

**Affiliations:** ^1^St. Mary Medical Center, Langhorne, PA 19047, USA

**Keywords:** da Vinci, Vesalius, Harvey, Galen, circulation

## Abstract

Ancient societies believed the heart was the most important organ in the body. Ancient religions held that only through the heart could one connect with God. During Europe’s Middle Ages there was little to no advances regarding the heart’s workings. As the Middle Ages gave way to the Renaissance, scientists and physicians began questioning long-standing theories on the heart. The first accurate descriptions of the heart and its function were written, and the first anatomically correct representations of the heart were drawn.

## 1. Introduction

Our ancient ancestors believed the heart was the most important organ in the body. Ancient societies elevated the heart to the position held today by the brain; body ruler, its source of power and warmth, and home to the soul [1]. Ancient religions held that only through the heart could one connect with God. During Europe’s Middle Ages there was little to no advances regarding the heart’s workings [2, 3]. In other parts of the world, especially in Arabic society, physician-scientists did start to better understand heart anatomy and function. But they were limited by laws preventing dissection and experimentation.

As the Middle Ages gave way to the Renaissance, scientists and physicians began questioning long-standing accepted theories on the heart. These were mainly the heart according to Galen, Aristotle and Hippocrates [2, 3].

**Fig. 1.**
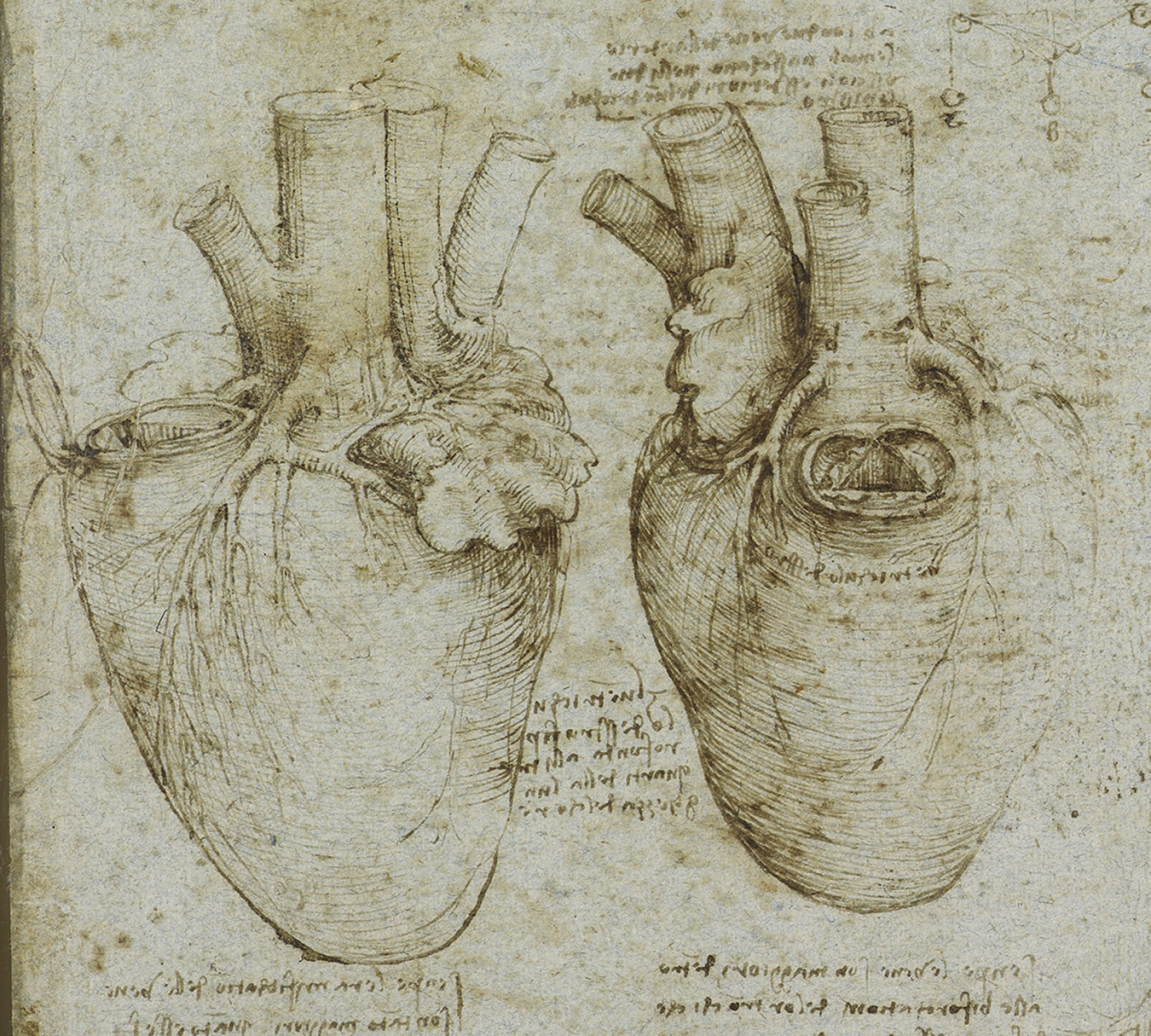
**Heart drawing and annotations by Leonardo da Vinci**. Source: Collection Windsor Castle, United Kingdom. Royal Collection Trust/© His Majesty King Charles III 2023.

Three giants stand out from the fifteenth through seventeenth centuries who first described the heart as we see it today; a painter, an anatomist, and a physician to kings. This heart renaissance forever changed how humans viewed the purpose of this beating organ inside them. Through examination and experimentation Leonardo Da Vinci, Andreas Vesalius, and William Harvey began to elucidate the heart’s anatomy and function. They sketched what we now accept as the first accurate anatomical representations of the heart (Fig. 1).

## 2. da Vinci

Leonardo da Vinci (b. 1452) preformed human dissections, sketching skin, tendons, muscles, and bones. Like many artists of the Renaissance (including Michelangelo), performing dissections helped them more accurately represent the body in their art. But da Vinci explored further into the body. As a result, he began to work with Marcantonio Della Torre, professor of anatomy at the University of Padua [4]. Della Torre intended to publish a book with da Vinci’s anatomical drawings, but died prematurely of plague in 1511.

Da Vinci, through detailed anatomical study, correctly understood the heart was four chambered. He established through experiments that Galen was wrong; blood, not air, entered the heart from the lungs. He demonstrated through experimentation that valves allowed the blood to flow in only one direction. Further, he experimentally determined that the aortic valve closed due to vortices. By filling an ox heart with wax, he recreated the aortic structure in glass. He pumped water containing grass seeds through the glass aorta and watched the seeds swirling back towards the valve. This observation was not demonstrated again until 1968 by Oxford engineers Brian and Francis Bellhouse. It was not until a year after they published their work when they found da Vinci had come to the same conclusion five hundred years earlier.

While da Vinci was heavily influenced by Galen’s (b. 129 CE) teachings, he did make new discoveries. For example, he determined the heart, not the liver (as Galen believed), pumped blood throughout the body. In Galen’s scheme, food in the gut was transported to the liver where blood was formed. The blood then flowed to the right ventricle where some entered the lungs via the pulmonary artery to nourish them, while the remainder reached the left ventricle through invisible pores in the interventricular septum. Supposedly, da Vinci held on to Galen’s view that blood moved from the right to left ventricle through pores in the interventricular septum. In fact, da Vinci did draw one small cartoon-like illustration of the heart’s interventricular septum, with lines drawn through it suggesting Galen’s “pores”. However, all of his anatomical drawings of the interventricular septum do not contain visible pores. It may be that early on, da Vinci accepted there may be septal pores per Galen’s theory; or he may have feared venturing against Church doctrine as noted by several historians [5].

Unlike Aristotle (b. 384 BCE), da Vinci believed the soul was not in the heart, but the brain—specifically above the optic chiasm in the anterior third ventricle. Here it resided in judgement where all senses came together; the *senso commune*, or common sense [6].

Da Vinci was the first to write that narrowing of coronary arteries can cause sudden death. In 1506, he observed a man supposedly 100-years-old die suddenly and peacefully. Da Vinci performed “an anatomy to discern the cause of a death so sweet” [7]. His dissection led him to discover a “thickened coat” in the coronaries, deducing this as the cause of the man’s sudden death.

No longer in the Middle Ages, Da Vinci’s revelations on the structure and function of the heart were the first real progress in Western understanding in over 1000 years [2, 3]. However, after dying, his works passed on to his apprentice Count Francesco Melzi. Melzi’s descendants sold Leonardo’s journals. Eventually purchased by English King Charles II, Da Vinci’s anatomical drawings and notes were forgotten in the Royal Library at Windsor; not rediscovered and published until 1796—more than 250 years after his death.

## 3. Vesalius

Andreas Vesalius (Latinized from Andries van Wezel; b. 1514) was a Flemish anatomist and physician. He left Belgium in 1533 to go to Padua, the center of Western science and medicine. Arriving, he found anatomy demonstrations had become more like circus theaters than knowledge centers. Anatomists were entertaining crowds with their knowledge acquired from Galen and the Greeks. Vesalius wanted to challenge old theories. But he needed cadavers.

Vesalius became one of history’s most expert body snatchers [8, 9]. He and his students would cut down criminals from gallows and remove half buried bodies from cemeteries. They would break into ossuaries and steal bodies. The more he dissected, the more he questioned accepted theories on the heart and body.

A Galen theory that bothered Vesalius was that blood moved through invisible pores from the right to left side of the heart [9]. Vesalius studied hearts and did not see pores, but instead a thick muscular wall separating the ventricles. Unfortunately, he did not take the next step and discover circulation. Vesalius did accept some of Galen’s erroneous theories, such as blood was produced by the liver and consumed in the body, and that the heart was a furnace [9].

**Fig. 2.**
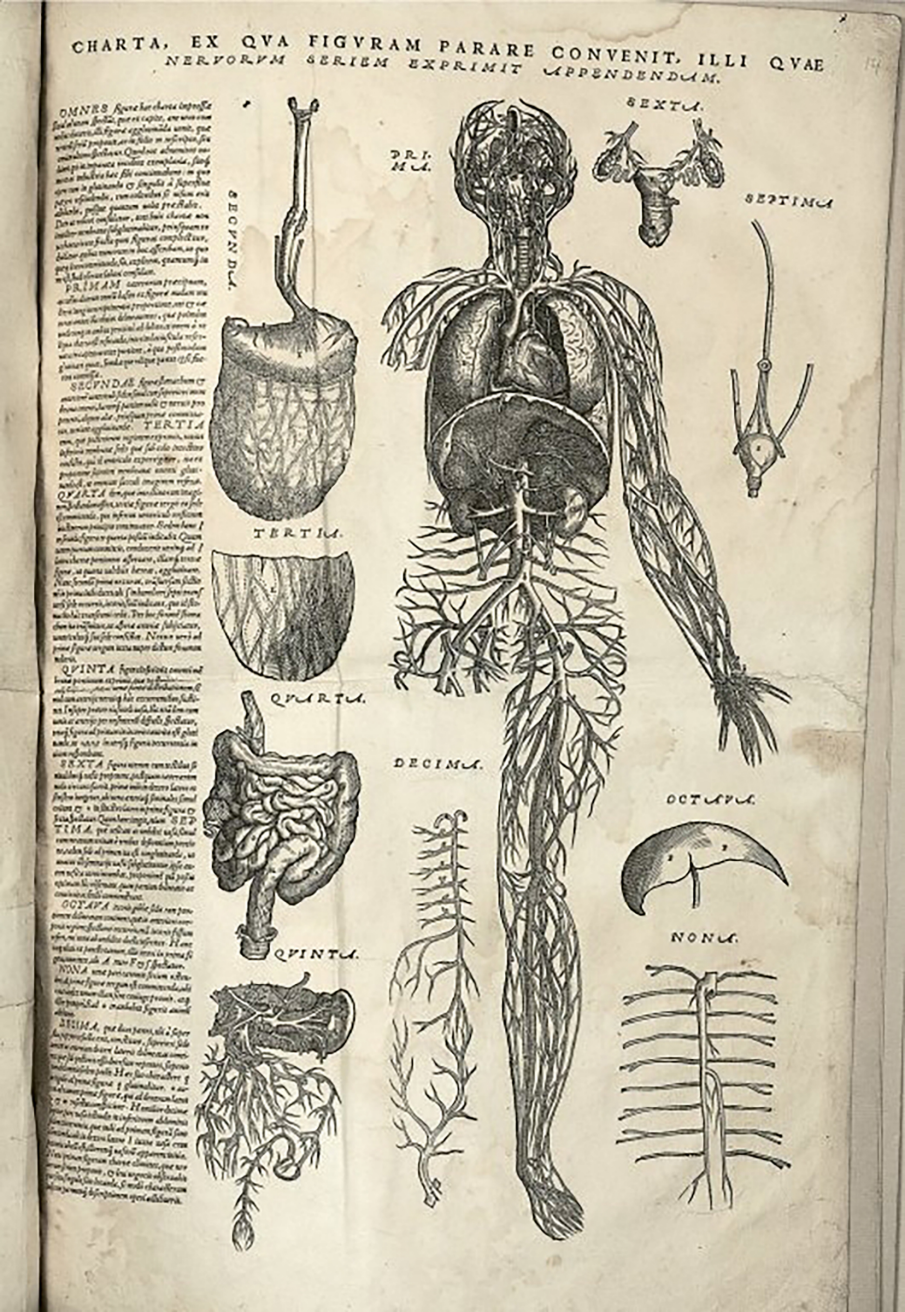
**Human anatomical chart of blood vessels, with heart, lungs, liver and kidneys included**. Other organs are numbered and arranged around it. Andreas Vesalius. De Humani Corporis Fabrica, 1543. Wikipedia Commons. Public Domain.

At age 28, Vesalius wrote one of the most important books in the history of medicine, De Humani Corporis Fabrica (On the Fabric of the Human Body) [9]. Published in 1543, Vesalius challenged much of what was known to date regarding the human body, correcting many of Galen’s and Aristotle’s errors. Some believe Vesalius hired Titian (or artists from Titian’s school) to illustrate his book (Fig. 2). He diagramed the paths of arteries and veins throughout the body. He also published the first drawings of valves in veins.

Historians speculate that if Vesalius had not left Padua to become personal physician to Charles V of Spain, he might have discovered circulation.

By the middle of the sixteenth century, physicians and scientists were questioning the Galenic heart. They were starting to understand heart anatomy and function. At the same time, Renaissance painters and poets began using the heart as a symbol of romantic love and love of God.

## 4. Harvey

William Harvey (b. 1578), son of a farmer, was personal physician to two English kings, James I and Charles I. As a medical student, Harvey considered Aristotle his master. Like Vesalius, Harvey went to Padua to study under Hieronymus Fabricius (discoverer of valves in veins). Based on his experiments, Harvey is credited as the first to mechanistically demonstrate circulation. For his circulation theory, Harvey had an advantage that Galen did not: the invention of mechanical pumps. By Harvey’s time, hydraulic water pumps for mining and fire-extinguishing were in common use. The metaphor was there for him to comprehend.

In 1628, Harvey wrote the *Anatomical Study of the Motion of the Heart and of the Blood in Animals * [10]. To disprove accepted Galenic theories, Harvey experimented. For example, he tied off a section of artery with two strings, and cutting it open found only blood inside, not air or spirits, as Galen had taught. He demonstrated when the pulmonary artery was ligated and the right ventricle filled with water, no fluid crossed the septum into the left ventricle through invisible pores [10, 11].

Harvey performed dissections on live animals and executed criminals to packed amphitheaters. He lectured in Latin, accompanied by lutes. He would cut open a dog’s pulmonary artery, showering the audience with blood as the right ventricle contracted—what fun [11]!

**Fig. 3.**
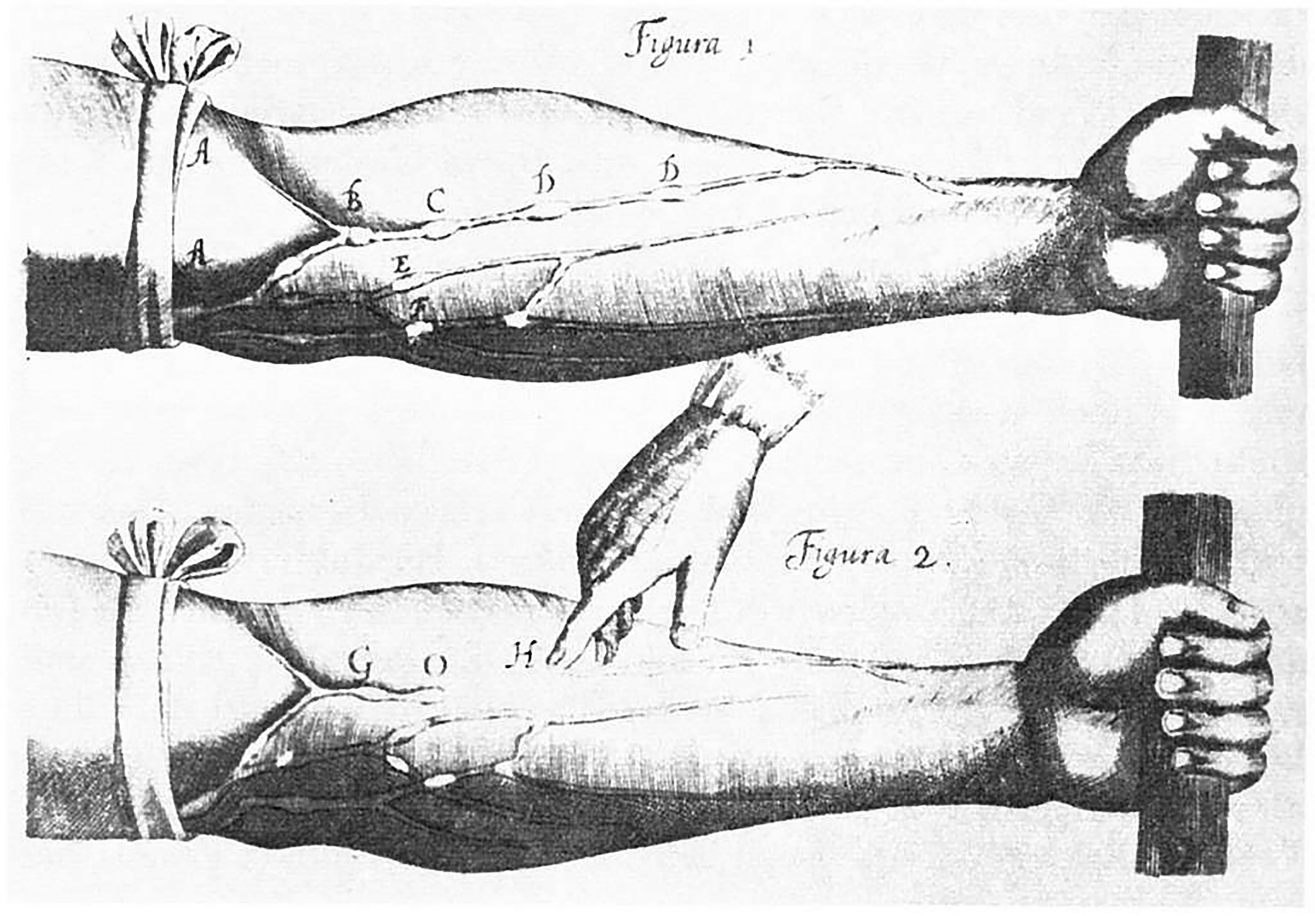
**Image from William Harvey’s Exercitatio Anatomica de Motu Cordis et Sanguinis in Animalibus, 1628, showing that the blood circulated**. When a vein was blocked with a tourniquet, it swelled up, the blood unable to escape back towards the heart. Sigerist, Henry E. (1965) Große Ärzte, München, Deutschland: J.F. Lehmans Verlag (5. Auflage) (1. Auflage 1958) plate 26 p 120. Wikipedia Commons. Public Domain.

To demonstrate his circulation theory, Harvey applied a tourniquet to an arm just tight enough to block blood flow through the veins, without affecting the muscular arteries (Fig. 3). When he did, the section of the arm below the tourniquet swelled, as would be expected if blood entered the arm but could exit. When he retightened the tourniquet further, blocking flow in both arteries and veins, blood did not build up in the veins and the arm did not swell. Additionally, blood built up in the arteries above the tight tourniquet. Thus, Harvey surmised blood moved “thither by the arteries, hither by the veins” [10]. Harvey theorized there were invisible pores, too small to see, connecting the two vascular systems (capillaries were not discovered until 1661 when Italian scientist Marcello Malpighi examined them in frog lungs using a microscope).

Harvey wrote: “It has been shown by reason and experiment that by the beat of the ventricles blood flows through the lungs and it is pumped to the whole body. There it passes through pores in the flesh into the veins through which it returns from the periphery…It must then be concluded that the blood in the animal body moves around in a circle continuously, and that the action or function of the heart is to accomplish this by pumping. This is the only reason for the motion and beat of the heart [10]”.

Alas, the heart was merely a pump. Yet, Harvey publicly stated the heart was the seat of emotions and did not challenge its metaphysical role (possibly out of fear for his life). He did believe the heart, near the physical center of the body, by virtue of this circulation, distributed warmth throughout the body.

Galen believed after food was ingested it converted into blood in the liver, then pumped out to the body. This theory is what most of Western civilization accepted for over 1000 years [2, 3]. Unknown to Harvey, and the world, da Vinci had already calculated the amount of blood pumped by each heartbeat in a day (7600 liters per day). Harvey came to the same conclusion. Therefore, blood had to be recirculated [10, 11].

## 5. Other Notables of the Heart Renaissance

Realdo Colombo (1516–1559), the Chair of Anatomy and Surgery at the University of Padua after Vesalius, correctly described the pulmonary circulation. He based his theory on the facts that the pulmonary vein was full of blood, which would not be the case if the vessel were constructed solely for conveying air and vapors; he was unable to demonstrate Galen’s pores in the interventricular septum; and he recognized that the heart valves were competent and thus vital blood cannot return to the lungs.

Colombo’s account of the pulmonary circuit was preceded by that of Michael Servetus (1511–1553), a Spanish philosopher-theologian, who published a treatise, Christianismi restitutio (1553), where he also challenged Galen’s theories and proposed that blood is driven from the right ventricle to the lungs, where it mingles with inspired air and is ultimately drawn into the left ventricle. However, because Servetus’s Christianismi restitutio was completely suppressed, as he was condemned by John Calvin to burn at the stake on a pyre of his own books, it is not known whether Colombo had read his work.

It is important to note that the pulmonary circulation had actually been described 300 years earlier by Arab physician Ibn al-Nafis (1213–1288). Al-Nafis disagreed with Galen that blood could pass through the interventricular septum, but that all blood that reached the left ventricle passed through the lungs. Unfortunately, al-Nafis’ work was not published in print until the early 20th century. It is not known whether Colombo or Servetus could have seen a translation of al-Nafis’ work.

Girolamo Fabrizio (also known as Hieronymus Fabricius, 1537–1619), who was a professor of anatomy at Padua when William Harvey studied there, identified venous valves in 1574 and published a description of them in 1603. Fabricius thought that the purpose of the venous valves was to prevent blood from pooling in the hands and feet. Harvey, his pupil, would later use these valves as evidence that blood in the veins can flow only towards the heart, and that therefore blood must circulate through the body, hither through the arteries and thither through the veins.

Cesare Cremonini (1550–1631), a professor of natural philosophy at Padua, developed a quantitative argument to support his theory that blood transported to the body through arteries was a major source of nutrition. This contradicted Galen’s theory that arteries were just vehicles for heat and spirit. He pointed out that arterial blood is diffused in great quantity. What becomes of it if it is always generated but not consumed as nutrient. Surely it would grow to infinity, he concluded.

Finally, Italian biologist and physician Marcello Malpighi (1628–1694) established the presence of capillaries connecting the arterial and venous trees in 1661. He examined arteries and veins in frog lungs with a new device called the ‘microscope’.

## 6. Conclusions

By the seventeenth century, anatomical knowledge of the heart was surprisingly accurate, and Harvey’s theories of a double circuit, made up of pulmonary and systemic circulations, became widely accepted. It was during the Renaissance that science changed forever our view of the heart. The heart was nothing more than a mechanical pump devoid of spiritual significance. Rene Descartes (b. 1596), one of the first to accept Harvey’s circulation theory, went a step further describing the heart as more akin to a machine-like furnace causing the blood to expand and rush throughout the body [12].

As the Renaissance advanced into the Age of Enlightenment, the heart was no longer considered the seat of the soul, but merely an organ which responded to emotions and feelings at the direction of the brain. Going forward the heart would only metaphorically represent love, but this metaphor would remain powerful [3].
